# Plasma and neutrophil fatty acid composition in advanced cancer patients and response to fish oil supplementation

**DOI:** 10.1038/sj.bjc.6600659

**Published:** 2002-12-02

**Authors:** V C Pratt, S Watanabe, E Bruera, J Mackey, M T Clandinin, V E Baracos, C J Field

**Affiliations:** Department of Agricultural, Food and Nutritional Sciences, University of Alberta, 410 Ag/Forestry Center, Edmonton, Alberta, T6G 2P5 Canada; Department of Oncology, Division of Palliative Care Medicine, University of Alberta, c/o Grey Nuns Community Hospital 1100 Youville Drive West Room 4324, Edmonton, Alberta Canada T6L 5X8; Department of Symptom Control and Palliative Care, Houston, Texas, USA, 1515 Holcombe Blvd. Box 8 Houston, Texas 77030 USA; Department of Oncology, University of Alberta, Cross Cancer Institute - 11560 University Avenue Edmonton, Alberta T6G 1Z2 Canada

**Keywords:** weight loss, neutrophil, clinical trial, phospholipids, chemotherapy, nutritional status

## Abstract

Metabolic demand and altered supply of essential nutrients is poorly characterised in patients with advanced cancer. A possible imbalance or deficiency of essential fatty acids is suggested by reported beneficial effects of fish oil supplementation. To assess fatty acid status (composition of plasma and neutrophil phospholipids) in advanced cancer patients before and after 14 days of supplementation (12±1 g day^−1^) with fish (eicosapentaenoic acid, and docosahexaenoic acid) or placebo (olive) oil. Blood was drawn from cancer patients experiencing weight loss of >5% body weight (*n*=23). Fatty acid composition of plasma phospholipids and the major phospholipid classes of isolated neutrophils were determined using gas liquid chromatography. At baseline, patients with advanced cancer exhibited low levels (<30% of normal values) of plasma phospholipids and constituent fatty acids and elevated 20 : 4 *n*-6 content in neutrophil phospholipids. High *n*-6/*n*-3 fatty acid ratios in neutrophil and plasma phospholipids were inversely related to body mass index. Fish oil supplementation raised eicosapentaenoic acid and docosahexaenoic acid content in plasma but not neutrophil phospholipids. 20 : 4 *n*-6 content was reduced in neutrophil PI following supplementation with fish oil. Change in body weight during the supplementation period related directly to increases in eicosapentaenoic acid in plasma. Advanced cancer patients have alterations in lipid metabolism potentially due to nutritional status and/or chemotherapy. Potential obstacles in fatty acid utilisation must be addressed in future trials aiming to improve outcomes using nutritional intervention with fish oils.

*British Journal of Cancer* (2002) **87**, 1370–1378. doi:10.1038/sj.bjc.6600659 www.bjcancer.com

© 2002 Cancer Research UK

## 

Dietary supplementation with the *n*-3 polyunsaturated fatty acids (PUFA), eicosapentanoic acid (EPA) and docosahexanoic acid (DHA) as either fish oil capsules or a fish oil- supplemented enteral formula has been reported to attenuate weight loss, increase appetite, improve quality of life and prolong survival in weight losing cancer patients (Wigmore *et al*, 1996, 1997, 2000; Barber *et al*, 1999). Beneficial effects of *n*-3 fatty acid supplementation imply the presence of an imbalance between essential *n*-6 and *n*-3 fatty acids or a deficiency of *n*-3 fatty acids. Indices of fatty acid status may have prognostic value for selecting patients likely to obtain benefits from supplementation, however, to date supplementation has not been prospectively examined based on determined fatty acid status. Detailed indices of fatty acid status are also of interest for assessing the efficacy of supplementation protocols.

The fatty acid status of patients with advanced cancer is not well characterised. There is some evidence for abnormalities in lipid metabolism, including increased lipolysis (Kitada *et al*, 1982; Taylor *et al*, 1992) and oxidation of free fatty acids, (Hansell *et al*, 1986; Douglas and Shaw, 1990) and hyper-triglyceridemia (Grunfeld and Feingold, 1996; Tisdale, 1999). Abnormalities of plasma lipid profiles of cancer patients compared to healthy individuals have been reported (Mcclinton *et al*, 1991). Furthermore, many drug agents used to treat cancer have been reported to influence lipid metabolism (Ara and Teicher, 1996).

Fatty acid composition of plasma phospholipid (PL) and different cell types (erythrocytes, neutrophils) reflect, respectively, short and long term patterns of dietary fatty acid consumption, and are frequently used as indices of fatty acid status (Clandinin *et al*, 1983; Stanford *et al*, 1991; Diboune *et al*, 1992; Pratt *et al*, 2001). Neutrophils are of particular interest as these cells have been implicated in several disease states with an inflammatory component (Sperling *et al*, 1993; Matzner, 1997; Matsukawa and Yoshinaga, 1998; Ambrosio and Tritto, 1999). Neutrophils secrete and respond to several cytokines involved in the wasting response of cancer (Cassatella *et al*, 1997) which are attenuated by dietary fish oils (Blok *et al*, 1996; Barber *et al*, 1998). Furthermore, major neutrophil functions are membrane dependent (Deitch, 1984; de Pablo and Alvarez de Cienfuegos, 2000). Therefore, the ability to alter the membrane composition of neutrophils by fish oil supplementation may have important implications for reducing inflammation and wasting in advanced cancer patients.

The objectives of this study were (1) to assess the fatty acid composition of plasma and neutrophil PL of patients with advanced cancer and to compare this with other patient groups and healthy subjects; (2) to assess *n*-6 and *n*-3 fatty acid content and extent of incorporation of supplemented oils into plasma and neutrophil PL in patients with advanced cancer, and (3) to identify relationships among *n*-6 and *n*-3 fatty acids in plasma and cell PL and some select parameters of nutritional status.

## MATERIALS AND METHODS

### Materials

H-plates and G-plates were purchased from Analtech (Newark, DE, USA). All solvents were purchased from VWR (Edmonton, AB, Canada). Bovine serum albumin (BSA, Fraction V), trypan blue, Ficoll Hypaque gradients, chemicals for buffers and all other lipid supplies, including standards, were purchased from Sigma Chemicals (St. Louis, MO, USA).

### Subjects

Protocols for study of all subjects were reviewed and approved by the Research Ethics Board of the Alberta Cancer Board or Faculty of Medicine Research Ethics Board of the University of Alberta. All subjects gave informed consent. Due to the paucity of reference data for fatty acid composition of plasma and immune cells in advanced cancer patients, multiple subject groups were selected for comparison. We compared patients with advanced cancer to victims of major burn injury, which characteristically have a very strong inflammatory reaction and marked changes in phospholipids (Pratt *et al*, 2001) as well as a group of breast cancer patients receiving high dose chemotherapy to assess the effect of chemotherapy on fatty acids. Each group of subjects had plasma and cellular PL compositions assessed using the same method in the same laboratory as the advanced cancer group. In the absence of a large base of reference data, these patient groups, which represent a wide range of patient groups from critically ill to healthy subjects, were used to obtain a context for the range of plasma PL fatty acids observed in advanced cancer patients in this study. A group of healthy subjects from the local population served as a control comparison to provide reference values for the healthy population as an age and sex matched control cohort was not feasible.

#### Advanced cancer

Patients with advanced cancer (defined as locally recurrent or metastatic) were recruited from the Acute Palliative Care Unit and Hospice, Caritas Health Group, and both hospitalised and outpatients at the Cross Cancer Institute (Edmonton, Alberta, Canada). Inclusion criteria included: (1) presence of anorexia, defined as >3 on a visual analogue scale; (2) loss of 5% or more of their pre-illness body weight; (3) ability to maintain oral intake, (4) normal cognition, defined as a normal Mini-Mental State examination (Periera *et al*, 1998) for age and level of education and (5) ability and willingness to give written, informed consent. Eligible participants were randomised using a computer-generated sequence and allocated to receive identical 1 g gelatin capsules containing fish oil (180 mg EPA and 120 mg DHA, Banner Pharmacaps, Olds, AB, Canada) or placebo (olive oil). Olive oil was selected as the placebo, as it was not expected to alter plasma lipids or essential fatty acid metabolism (Thien *et al*, 1993; Yaqoob *et al*, 2000). The fatty acid composition of the fish oil and placebo capsules was determined using gas liquid chromatography (Layne *et al*, 1996) (Table 1

**Table 1:**
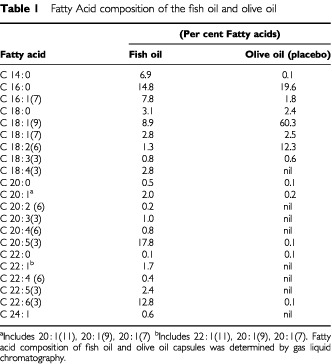
Fatty Acid composition of the fish oil and olive oil

). All investigators and health care workers were blinded to the treatment received throughout the trial.

Subjects were instructed to take 18 capsules orally each day for 14 days. On day 0 and day 14 of supplementation, a non-fasting sample of peripheral blood (16 mL) was drawn by a registered nurse during in-home visits or in the clinic. Weight (without shoes and wearing light clothes) was obtained at day 1 and day 14. On day 1, height and pre-illness stable weight was also documented. Patients were instructed by a research assistant to keep a 3-day food day record during three consecutive days both at the beginning and end of the 14 days supplementation period. The nutrient content of coded food items was estimated using Food Processor II nutrient analysis programme with the Canadian Nutrient Data File (Esha Research, Salem, Oregon (Bruera *et al*, 1986).

The patients with advanced cancer were a cohort of subjects participating in a larger study (Bruera *et al*, 2002). A random sub-sample of 14 subjects per treatment arm were assigned to receive more detailed measures of fatty acid status described here. The number of patients entered into the study was based on the ability to detect a change at a 5% significance level in *n*-3 fatty acids in plasma after 2 weeks of supplementation with fish oil. The characteristics of the subjects are shown (Table 2

**Table 2:**
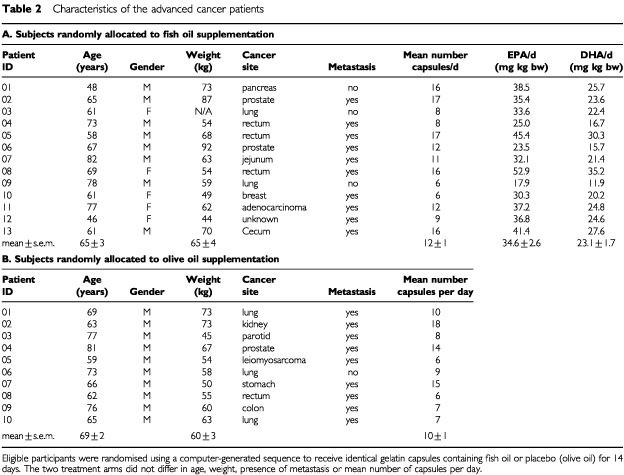
Characteristics of the advanced cancer patients

).

#### Burn injury

Patients and determinations are described elsewhere in detail (Pratt *et al*, 2001). Ten adult patients were recruited who had an age range from 20 to 65 years (mean=40±4 years). The range of burn size was from 12–90% (mean=37±5% total body surface area). A non-fasting venous blood sample (10 mL) was collected within the first 12 days (*n*=10) and after 50 days (*n*=8) following the burn injury. Timepoints were compared using a repeated measures ANOVA.

#### High dose chemotherapy with stem cell transplant

Three women, ages 35–47, with high risk stage II and III breast cancer at the Cross Cancer Institute (Edmonton, Alberta), were treated with high-dose chemotherapy with autologous stem cell transplant (Mccauley, 1996). Briefly, patients underwent four 21 days cycles of intravenously administered induction chemotherapy (5-fluorouracil, adriamycin and cyclophosphamide). Peripheral blood stem cells were harvested on day 12 after the third induction chemotherapy cycle. Approximately 3 weeks after the final induction treatment, patients were admitted for administration of intensive, high-dose chemotherapy (HDCT) consisting of cyclophosphamide (6 g m^−2^), mitoxantrone (64 mg m^−2^) and vinorelbine (95 mg m^−2^) over 4 days. After HDCT, they were transplanted with autologous stem cells harvested earlier (Nabholtz *et al*, 1997). Reconstitution was defined to be clinically complete when white blood cell counts returned to at least 2×10^9^ L^−1^ and platelet counts to at least 50×10^9^ L^−1^ (Ho *et al*, 1994). The women did not have immunosuppressive or autoimmune disease, or other types of cancer, and had a body mass index (BMI) between 21–27. Blood (16 mL) was collected into heparinised tubes at stem cell harvest (∼2 weeks post induction chemotherapy) and on the day of discharge (∼2 weeks post-HDCT). A paired *t*-test was used to compare pre- and post chemotherapy values.

#### Healthy subjects

Blood was sampled from healthy subjects (*n*=6) to serve as a reference for neutrophil and plasma PL composition. The age range of healthy subjects was 21 to 59 years and all of the subjects had BMI <25.

### Isolation of plasma and neutrophils

Fatty acid composition was assessed on neutrophils isolated using Ficoll Hypaque gradient centrifugation (Boyum, 1968). Plasma was removed from the top of the gradient and frozen immediately at −70°C. The neutrophil band was removed and cells washed twice with Krebs-Ringer HEPES (KRH) buffer with BSA (5 g L^−1^). Cells were cultured in complete culture media and cell viability was assessed at greater than 99% for all samples. Cells were pelleted and frozen immediately at −70°C until lipid analysis.

### Fatty acid analysis of neutrophil and plasma PL

#### Neutrophil fatty acid composition

A modified Folch method was used to extract lipids from isolated neutrophils (Field *et al*, 1989). Composition of individual PL ((phosphatidylcholine (PC), phosphatidylethanolamine (PE), phosphatidylinositol (PI), and phosphatidylserine (PS)) fatty acid methyl esters was determined using gas liquid chromatography as previously described (Pratt *et al*, 2002). Peaks of fatty acid methyl esters were identified by comparisons with standards purchased from Supelco (Bellefonte, PA, USA) and Sigma Chemical (St. Louis, MO, USA). Per cent of saturated (SFA), monounsaturated (MUFA), polyunsaturated (PUFA), 20 : 4 *n*-6, total *n*-6 and *n*-3 fatty acids and *n*-6/*n*-3 ratios are presented.

#### Plasma fatty acid PL

Plasma PL fatty acid composition was determined as previously described in detail (Pratt *et al*, 2001). Briefly, plasma (100 μl) fatty acids were extracted using chloroform/methanol and serum PL isolated on G-plates (Layne *et al*, 1996). For quantitative analysis, C17:0 standard was added to the scraped serum PL (10 μg of C17:0) followed by direct methylation. Fatty acid methyl esters separated by automated gas liquid chromatography as described above. Total PL content of individual fatty acids was calculated on both a quantitative and per cent (w w^−1^) basis (Pratt *et al*, 2001).

### Statistical analysis

Data are reported as mean±s.e.m. Variations in baseline and post-supplementation fatty acid compositions of plasma and neutrophil PL were analysed using BMI to stratify the subjects into high and low groups (subjects with values above and below the overall group mean for body mass index). A *t*-test was used to assess differences between the high and low groups. Specific correlations between BMI, kcal kg^−1^ BW intake against plasma PL were performed. To measure changes in fatty acid composition within a supplementation group, a paired *t*-test was used. Differences between groups and timepoints were analysed using repeated measures ANOVA. Significant differences (*P*<0.05) between treatments and day 0 and 14 were identified using least square means. All statistical analyses were conducted using the SAS statistical package (Version 6.12, SAS Institute, Cary, NC, USA).

## RESULTS

### Subjects

Thirteen and 10 subjects completed 14 days of fish oil and placebo supplementation, respectively (Table 2) a total of 23 subjects. Self-reported intakes of capsules did not differ significantly between treatments. Reasons for not completing the study included failure to maintain intake of the oil capsules, complications due to cancer progression, and death (*n*=4). Groups did not differ significantly in age, body weight or the presence of metastasis. The primary site of the cancer varied widely among subjects in both groups (Table 2).

### Baseline comparisons to reference groups

#### Plasma PL

The baseline (pre-supplementation) levels and fatty acid composition of plasma PL for the 23 advanced cancer patients were contrasted with the other patient groups and healthy controls (Table 3

**Table 3:**
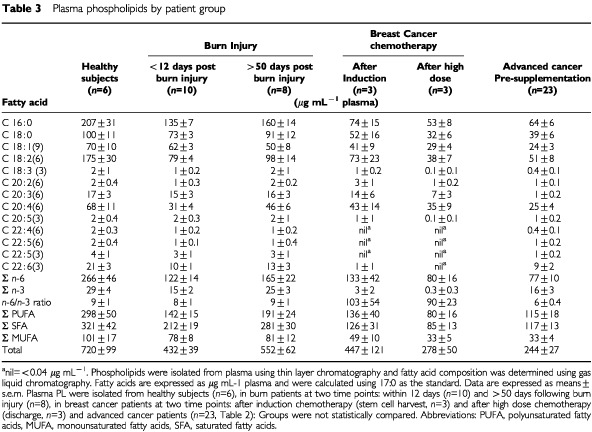
Plasma phospholipids by patient group

). Chemotherapy and burn patient groups were not compared statistically to the advanced cancer patients because they were not controlled cohorts for sex, age or body composition. The most striking observation from this analysis was that total plasma PL and most individual fatty acids in the PL of advanced cancer patients, including the essential fatty acids, 18 : 2 *n*-6 and 18 : 3 *n*-3, were the lowest of all groups studied at ∼30% of the levels observed in healthy subjects (Table 3). Of the long chain polyunsaturated fatty acids (PUFA), only EPA levels were relatively well-maintained (93% of healthy controls) in the advanced cancer group. The ratio of mead acid (20 : 3 *n*-9) to 20 : 4 *n*-6, a measure of essential fatty acid deficiencies (Spector, 2000), was elevated in the advanced cancer patients (0.3±0.04) compared to healthy subjects (mean=0.01±0.0). Twelve days after major burn injury, levels of total plasma PL and most fatty acids therein were intermediate between healthy subjects and patients with advanced cancer and at 7 weeks postburn, plasma PL increased towards levels seen in healthy controls (Pratt *et al*, 2001). The chemotherapy group fell intermediate between the levels seen in healthy subjects and the patients with advanced cancer for most fatty acids. The most striking feature of the fatty acid profiles of the chemotherapy patients was the very low levels of long chain (>20 carbon long) PUFA. After induction chemotherapy, all PUFA with 22 carbons in chain length, with the exception of EPA and DHA, were below the limit of detection (Table 3). After high dose chemotherapy, DHA became undetectable and EPA levels fell to 0.1 μg mL^−1^ or about 7% of control values. Fatty acids of the *n*-6 series (18 : 2 *n*-6 and 20 : 3 *n*-6) were also strikingly low after high dose chemotherapy (22 and 41%, respectively, of healthy reference values).

#### Neutrophil PL

The fatty acid compositions of PC, PE, PI and PS was determined on isolated neutrophils. Data is shown in Table 4

**Table 4:**
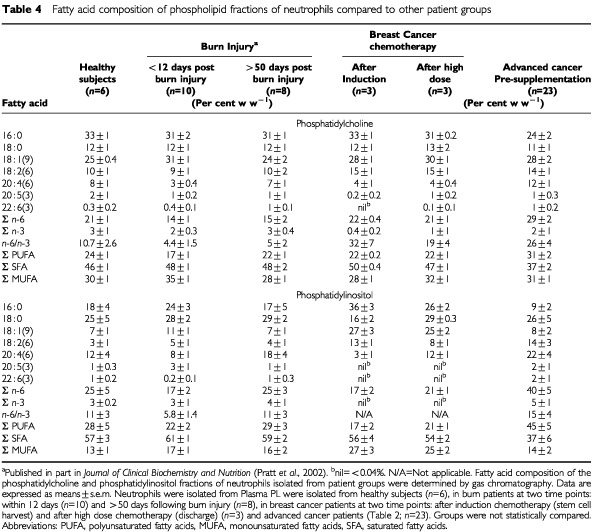
Fatty acid composition of phospholipid fractions of neutrophils compared to other patient groups

for PC, which comprises the largest proportion (∼39%) of neutrophil PL (Gottfried, 1972), and PI, which is reported to play a role in the signalling of the neutrophil oxidative burst (Cockcroft and Stutchfield, 1989; Hundt and Schmidt, 1992; Sayeed, 1998). Higher levels of 20 : 4 *n*-6 in all PL classes of neutrophils (+80% in PI, +80% in PC, +22% in PE and +100% in PS) contributed to greater PUFA content and a higher *n*-6/*n*-3 fatty acid ratio in advanced cancer patients compared to other patient groups and healthy subjects (Table 4). This elevated PUFA content was matched with lower saturated fatty acid content, primarily 16 : 0, whereas content of monounsaturated fatty acids did not appear different than other groups. Chemotherapy patients had undetectable levels of *n*-3 fatty acids in neutrophil PI after induction chemotherapy and remained so after HDCT.

### Variation of fatty acid levels in patients with advanced cancer

At baseline (pre-supplementation) patients with advanced cancer appeared different than other patient groups in fatty acid compositions of PL in plasma and neutrophils. There was also considerable variation within the advanced cancer patient population for the studied variables. We sought to explain some of this variation by relating the compositions of plasma and cell PL to nutritional status to interpret the data obtained here and to aid in the design of future studies.

#### Measures of nutritional status

BMI, total caloric intake, and fat intake at the time of entry into the study were chosen as measures of nutritional status. The mean percentage of calories derived from fat was 31±7%. Caloric intake and total fat intake were highly correlated (*r*=0.97, *P*=0.0001), therefore, caloric intake was used in subsequent analysis. Both BMI and caloric intake varied over a broad range and were not significantly correlated (*P*=0.14).

**Figure 1:**
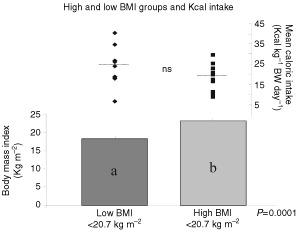
Advanced cancer patients were stratified at baseline into high and low BMI groups based on the group mean (20.7 kg m^−2^; *n*=23). Subjects with BMIs above the group mean represent the low BMI group (*n*=12) and subjects with BMIs above the group mean comprise the high BMI group (*n*=11). Columns represent mean±s.e.m. of the high and low BMI groups. The range of mean caloric intakes (days 1, 2 and 3 of supplementation) within each BMI grouping are shown for each patient using data points. Caloric intakes were estimated using 3 day food records and analysed using the Food Processor II Nutrient Analysis Program with the Canadian Nutrient Data File. Significant differences (*P*<0.05) between BMI groupings and corresponding caloric intakes were detected using a *t*-test.

The average BMI of the 23 subjects with advanced cancer was 20.7 kg m^−2^ with a range of 15 to 28 kg m^−2^, representing a mixture of patients who would be regarded as severely underweight, underweight, within the normal range and even overweight. Subjects were divided into two groups with BMI above (high BMI) and below the overall group mean (low BMI; Figure 1

). Caloric intake ranged from 488 to 2152 kcal day^−1^ with one third (28%) of the subjects having intakes of <1000 kcal day^−1^.

Mean intakes did not differ significantly between BMI groups (low BMI=25±8 kcal kg^−1^ BW per day; high BMI=19±2 kcal kg^−1^ BW per day; Figure 1).

#### Fatty acids in plasma and neutrophil PL

Although the average levels of EPA and DHA in neutrophil membrane PL did not appear different from other patient groups, the content of these fatty acids were highly variable amongst patients with advanced cancer. Some subjects had undetectable levels and others had up to 4% EPA or DHA in either their PC or PI fractions. Total caloric intake did not explain variation in plasma or cell PL fatty acid levels. Patients with advanced cancer had the highest levels of *n*-6 fatty acids in neutrophil PL (see Table 4) compared to all other subjects and this was most marked in the patients with lower BMI. This was primarily due to the low BMI group having significantly greater 20 : 4 *n*-6 content in PC (16.0±2.0%) compared with the high BMI group (9.7±2.3%; *P*=0.05) and for PI, where patients in the low BMI group had twice the levels of 20 : 4 *n*-6 in PI (22.6±4.3%), compared to the high BMI group (10.7±6.2%; *P*=0.05). BMI and levels of 20 : 4 *n*-6 in neutrophil PI were inversely correlated (*r*=−0.61, *P*=0.02).

Neutrophils have been reported to lack delta-6-desaturase and are dependent on incorporation of PUFA from plasma PL (Chilton-Lopez *et al*, 1996; Barham *et al*, 2000), therefore, it would be expected that cell and plasma PL PUFA levels would be related. These relationships were strongest for total and individual *n*-3 fatty acid levels. The total *n*-3 fatty acid content of neutrophil PC was directly related to plasma PL *n*-3 fatty acid content (*r*=0.78; *P*=0.001). By contrast, the 18 : 2 *n*-6 and 20 : 4 *n*-6 content of neutrophil PL were not significantly related to plasma PL levels of these fatty acids. As would be expected based on the preceding data, the *n*-6/*n*-3 ratio of plasma PL was directly related to the *n*-6/*n*-3 ratio in neutrophil PC (*r*=0.67; *P*=0.007). The 20 : 3 *n*-9/20 : 4 *n*-6 ratio in plasma PL of advanced cancer patients did not differ between the high and low BMI groups (mean=0.3±0.04).

### Effects of fish oil and olive oil supplementation

#### Fatty acid composition of plasma PL

Subjects consuming olive oil for 2 weeks did not exhibit significant changes in plasma PL fatty acid composition compared with pre-supplementation values (data not shown). Neither the fish oil nor placebo group demonstrated significant increases in plasma total PL concentrations over the 2-week supplementation period (not shown). After supplementation with fish oil, there was a significant increase in the fraction of EPA (+2–34 μg ml^−1^) and DHA (+2–14 μg ml^−1^) in plasma PL (Table 5

**Table 5:**
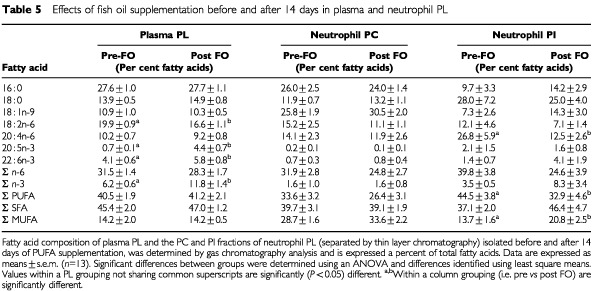
Effects of fish oil supplementation before and after 14 days in plasma and neutrophil PL

). Fish oil supplementation also resulted in a 15% reduction in 18 : 2 *n*-6 but did not change the 20 : 4 *n*-6 content of plasma (Table 5). After 14 days, the ratio of 20 : 3 *n*-9/20 : 4 *n*-6 was significantly different between fish oil and placebo groups (0.18±0.01 *vs* 0.27±0.04; *P*=0.03). The change in body weight gain during the 2 week supplementation period was directly related to the increase in EPA content in plasma PL (*r*=0.86; *P*=0.006; Figure 2

**Figure 2:**
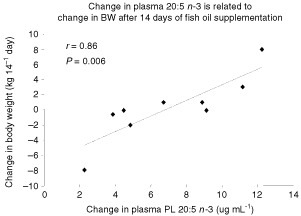
Data points represent the change in body weight from day 1 to day 14 of fish oil supplementation and the change in plasma EPA for subjects in the fish oil group (*n*=9). Body weight at either days 1 or 14 was not available for the four subjects missing in the analysis. There was a significant positive relationship between change in body weight and the change in plasma EPA.

).

#### Fatty acid composition of neutrophil PL

The fatty acid composition of neutrophil PL classes was not affected by olive oil supplementation (data not shown) and largely unaffected by fish oil supplementation. It is of note that in spite of a large elevation of plasma levels of EPA with fish oil treatment (Table 5), there was no parallel change in the EPA and DHA content of neutrophil membranes, nor in the amount of total *n*-3 fatty acids (Table 5). The only significant effect of fish oil supplementation on neutrophil PL fatty acid composition was a reduction in 20 : 4 *n*-6 content in the PI fraction (Table 5) to levels similar to control subjects. In PC, PE and PS there was a partial reduction of 20 : 4 *n*-6 levels, but this was not statistically significant.

## DISCUSSION

We made a comprehensive assessment of plasma and neutrophil PL fatty acids in a mixed group of advanced cancer patients experiencing weight loss. We observed very low levels of plasma total PL and unusual fatty acid content of neutrophil membrane PL that was marked by elevated levels of 20 : 4 *n*-6. Oral supplementation with fish oil for 2 weeks caused a significant increase in plasma PL levels of EPA and DHA, but did not alter total PL concentration in plasma nor did it raise *n*-3 fatty acid content in neutrophil PL. These results suggest multiple alterations in metabolism of lipids and specific fatty acids in cancer patients. While the causes of fatty acid abnormalities in advanced cancer have not been clearly characterised, the findings in this preliminary study in a small group of advanced cancer patients advance current understanding by outlining relationships linking fatty acid status and weight loss, as well as identifying potential causes of altered fatty acid profiles and obstacles that may impede lipid supplementation.

There is very little available data on the fatty acid status of patients with advanced cancer. Our context for comparison included healthy adults as well as additional patient groups from the same local population who had major burn injury (inflammatory injury; Tredget and Yu, 1992; Foex and Shelly, 1996) or were undergoing chemotherapy. The results presented here represent an initial approach and it is necessary to employ caution in comparing the data obtained for cancer patients with the other patient groups which serve mainly to place the advanced cancer patients in a context for discussion. Within the limitations of these comparisons, a number of points merit attention. Firstly, patients with advanced cancer had very low levels of plasma PL. Other studies on lipid supplementation reported the fraction of *n*-3 fatty acids in plasma PL, but not the plasma PL levels (Wigmore *et al*, 1996, 1997; Barber *et al*, 1999), so this abnormality was previously undetected. The cause(s) or the point in time that low levels of plasma PL evolved has not been identified, therefore it would seem important to follow this variable in newly diagnosed patients over the disease trajectory in further studies. Our data suggests chemotherapy as one possible cause for low levels of plasma PL. The inflammatory response, reported to occur in advanced cancer (Tisdale, 1999), may also contribute to reduced levels of plasma PL as patients with major burn injury had low plasma PL (Pratt *et al*, 2001) although to a lesser extent than the advanced cancer patients. High density lipoproteins (HDL) and low density lipoproteins (LDL), which account for the majority of total PL in plasma, have been recently reported to be reduced in a large, mixed group of cancer patients compared to non-cancer subjects (Fiorenza *et al*, 2000), and these may account in part for the low levels of total PL observed here. Both HDL and LDL levels would be affected by a proximal deficit in lipid absorption or of chylomicron or VLDL synthesis, and studies of lipid incorporation in advanced cancer patients would appear warranted.

A second unusual observation was high *n*-6/*n*-3 fatty acid ratios in plasma and cell PL of patients with advanced cancer, with an especially high fraction of 20 : 4 *n*-6 in all PL classes of neutrophils. It is not clear whether altered diet or altered metabolism of fatty acids would account for these differences, nor is it known to what extent the disease and therapy might have contributed. Subjects with the lowest BMIs exhibited higher levels of *n*-6 fatty acids in neutrophil PL and higher *n*-6/*n*-3 ratios in both plasma and neutrophil PL. This observation is consistent with the rationale given for *n*-3 fatty acid supplementation; i.e. that *n*-6 fatty acids are permissive for the action of catabolic mediators (Barber, 2001). However, while high levels of *n*-6 fatty acids were associated with BMI, it is not clear whether this was a cause or a consequence of the wasting process. BMI is commonly used to stratify patients in trials of anorexia/cachexia (Bruera *et al*, 1999; Loprinzi *et al*, 1999). The ratios of *n*-6/*n*-3 fatty acids may also be a useful criterion for stratification for future trials of fish oil supplementation. Our data suggests chemotherapy as one possible cause for altered fatty acid composition, especially depletion of PUFA. Although preliminary, the most striking feature of the fatty acid profiles of the chemotherapy patients was the very low levels of long chain PUFA (both *n*-6 and *n*-3 fatty acids) in plasma and cell PL. The *n*-6/*n*-3 ratios were considerably skewed by chemotherapy treatments, with the polyunsaturated *n*-3 fatty acids being most strongly affected. These changes were observed after induction chemotherapy and exhibited further declines after a high dose chemotherapy regimen, both of which employed widely used agents. Cytotoxic agents have been reported to interfere with the metabolism of PUFA (Marra *et al*, 1986) and may limit the endogenous production of EPA and DHA from 18 : 3 *n*-3 and of 20 : 4 *n*-6 from 18 : 2 *n*-6. This may support an argument to attempt to increase PUFA levels prior to chemotherapy or to include supplementation with post-chemotherapy re-alimentation.

We selected 14 days of supplementation, as this length of time was thought to support a high compliance and low drop out rate in a relatively fragile patient group. The efficacy of fatty acid incorporation during our short- term fish oil supplementation may be compared with related studies. Other authors employing fish oil capsules (Barber *et al*, 1998; Gogos *et al*, 1998) or fish oil – enriched enteral formula (Barber, 2001) to deliver similar amounts of fish oil to those reported here, report comparable elevations of per cent *n*-3 fatty acids in plasma PL of advanced cancer patients using longer intervention periods (Wigmore *et al*, 1997; Barber *et al*, 1999). In the present study, the ratio of 20 : 3 *n*-9 to 20 : 4 *n*-6 in plasma PL was reduced following the fish oil treatment and may suggest an improvement in essential fatty acid status. All patients allocated to receive fish oil exhibited increased levels of EPA and DHA in plasma PL, however, we observed poor correspondence between the intake of capsules reported by our patients and the magnitude of alterations in EPA and DHA in their plasma PL. It is unlikely that this discrepancy can be explained entirely by inaccurate reporting of capsules ingested. It is more likely that heterogeneity exists amongst patients in fatty acid digestion, absorption and incorporation into plasma PL. Given the heterogeneity of the individual patient response of plasma PL EPA, it is perhaps not surprising that the main study (Bruera *et al*, 2002) did not detect a significant treatment effect on weight or body composition. However, in this smaller subgroup of patients, the magnitude of the change in plasma PL EPA, was significantly related to the amount of weight gained by the subjects receiving fish oil, providing evidence for a dose response relationship. These results underline the importance of plasma PL fatty acid levels as a primary measure in oil supplementation studies.

Feeding fish oil to cancer patients for a short period can alter the fatty acid composition of peripheral immune cells. Supplementation did not raise *n*-3 fatty acid levels, however we did observe significant reductions in 20 : 4 *n*-6 in the PI fraction of neutrophils. PI is linked to a major receptor binding site (Hundt and Schmidt, 1992) important in intracellular signalling and activation (Tissot *et al*, 1991). Increasing the *n*-3 fatty acid composition of PI has been reported to be one mechanism for attenuation of pro-inflammatory responses (Sperling *et al*, 1993). Elevations in *n*-3 fatty acids accompanied by reductions in 20 : 4 *n*-6, in the PC and PE fractions of neutrophils has been reported after 3 weeks of fish oil supplementation in healthy volunteers consuming larger daily doses of fish oil (9.4 g EPA and 5 g DHA; Sperling *et al*, 1993). It is unclear why we did not see any increase in EPA or DHA in neutrophil PL, in spite of significantly increased levels of *n*-3 fatty acids in plasma, as changes have been observed within 14 days in healthy subjects (Lee *et al*, 1985; Tiwary *et al*, 1987; Chapkin and Carmichael, 1990; Sperling *et al*, 1993). The low concentrations of plasma PL may have limited the availability and subsequent incorporation of these fatty acids into cell membranes (Chapkin *et al*, 1988) or supplemented fatty acids may have been directed to oxidative pathways (Korber *et al*, 1999; Zuijdgeest-van Leeuwan *et al*, 2000) rather than incorporated into cellular membranes. The capacity to impact on immune functions by altering membrane fluidity, enzyme activities and intracellular signals (Traill and Wick, 1986; Sakata *et al*, 1987; Graber *et al*, 1994; Peck, 1994b; de Pablo and Alvarez de Cienfuegos, 2000) through nutritional supplementation is assumed to be dependent on incorporation of supplemented fatty acids into membranes (Russell *et al*, 1987; Kinsella, 1990; Lester, 1990; Peck, 1994a) and the optimal conditions of supplementation for this to occur are presently unknown. Therefore, obstacles exist that must be addressed in future trials aiming to alter immune functions through lipid supplementation.

At whatever time fatty acid supplementation may be contemplated, there are important issues pertaining to dose and delivery. Provision of high doses of fish oil and purified EPA may be limited by untoward gastrointestinal side effects, which, in some cases, may be managed by dose reduction or pancreatic enzyme supplements (Barber, 2001). Other limiting factors may include pancreatic secretions, absorption of fatty acids, biosynthesis of chylomicron lipid and apoproteins in enterocytes, hepatic uptake of chylomicron remnants and formation of lipid and apoproteins of VLDL, lipoprotein metabolism in blood and transfer of lipid constituents to cells. It would seem critical to determine what impediment(s) exist to propose timely and effective nutritional supplementation. Assessment of fatty acid status and lipid metabolism from the time of diagnosis and throughout the progression of different tumour types and characteristic therapies will provide valuable information regarding the timing of supplementation. Further studies investigating the metabolism of *n*-3 fatty acids in advanced cancer patients are warranted.
